# Departure time influences foraging associations in little penguins

**DOI:** 10.1371/journal.pone.0182734

**Published:** 2017-08-23

**Authors:** Grace J. Sutton, Andrew J. Hoskins, Maud Berlincourt, John P. Y. Arnould

**Affiliations:** 1 Deakin University, School of Life and Environmental Sciences (Burwood Campus), Victoria, Australia; 2 CSIRO Land and Water, Canberra, Australian Capital Territory, Australia; University of California Santa Cruz, UNITED STATES

## Abstract

Recent studies have documented that little penguins (*Eudyptula minor*) associate at sea, displaying synchronised diving behaviour throughout a foraging trip. However, previous observations were limited to a single foraging trip where only a small number of individuals were simultaneously tracked. Consequently, it is not known whether coordinated behaviour is consistent over time, or what factors influence it. In the present study, breeding adults were concurrently instrumented with GPS and dive behaviour data loggers for at least 2 consecutive foraging trips during guard and post-guard stage at two breeding colonies (London Bridge and Gabo Island, south-eastern Australia) of contrasting population size (approximately 100 and 30,000–40,000, respectively). At both colonies, individuals were sampled in areas of comparable nesting density and spatial area. At London Bridge, where individuals use a short (23 m) common pathway from their nests to the shoreline, > 90% (n = 42) of birds displayed foraging associations and 53–60% (n = 20) maintained temporally consistent associations with the same conspecifics. Neither intrinsic (sex, size or body condition) nor extrinsic (nest proximity) factors were found to influence foraging associations. However, individuals that departed from the colony at a similar time were more likely to associate during a foraging trip. At Gabo Island, where individuals use a longer (116 m) pathway with numerous tributaries to reach the shoreline, few individuals (< 31%; n = 13) from neighbouring nests associated at sea and only 1% (n = 1) maintained associations over subsequent trips. However, data from animal-borne video cameras indicated individuals at this colony displayed foraging associations of similar group size to those at London Bridge. This study reveals that group foraging behaviour occurs at multiple colonies and the pathways these individuals traverse with conspecifics may facilitate opportunistic group formation and resulting in foraging associations irrespective of nesting proximity and other factors.

## Introduction

In a highly dynamic environment, predators are faced with two main foraging pressures: finding prey before starving; and the metabolic cost of capturing and processing prey [1]. Animals may develop strategies to limit these pressures in order to optimise the energetic return of a foraging period [2]. For example, group foraging strategies are one method of reducing costs to the individuals by increasing the efficiency at which patches are discovered [3]. Furthermore, group foraging can assist in the capture of large prey, where individuals coordinate efforts to subdue prey [4], and small prey, where individuals further concentrate aggregations of prey, thereby increasing capture capacity and reducing metabolic costs [5,6].

Individuals may aggregate at a shared resource by detection of explicit cues presented by conspecifics at a prey patch (*local enhancement*) or may gain prey information from conspecifics at a communal roost (*information centre hypothesis*) [7,8]. Prey abundance and distribution plays an important role in the formation and maintenance of foraging groups [9,10]. When prey is unpredictably distributed, large foraging groups encounter more opportunities due to an increased ability to detect prey. However, within-group competition may decrease the net prey intake per individual when prey is limited [11]. In such instances, where group size exceeds the optimal foraging limit, competition for resources may outweigh the benefits of group foraging, which can lead to the dissolution of coalitions [12,13]. A degree of sociality is required between conspecifics for individuals to cooperate in groups.

Most penguin species are colonial breeders, which makes some social interaction between conspecifics inevitable. They are visual predators, consuming small schooling prey such as Clupeiformes (e.g. Anchovy, *Engraulis* sp. and Pilchard, *Sardinops* sp.), crustaceans (e.g. Krill, *Nyctiphanes* sp.*)*, cephalopods (e.g. Squid, *Nototodarus* sp.) [14–18], and, most recently detected, Cnidarians (e.g. Lion’s mane jellyfish, *Cyanea capillata*) [19,20]. Group association behaviour has been described in some penguin species [19,21–23] but it is not known to what degree they employ group foraging strategies.

The little penguin (*Eudyptula minor*) is the smallest of the penguin species, with a breeding distribution restricted to southern Australia and New Zealand [24]. During the breeding season, individuals are central place foragers and must return to the colony to feed their offspring [25,26]. The breeding season is highly influenced by local environmental conditions [27]. In good years, a successful breeding pair may raise 2 clutches, each consisting of 1 or 2 chicks [25]. During the early stages of chick rearing, or guard phase, one adult must attend the nest at all times. Once the chicks are larger, usually after 10–14 days old, they can be left alone while both adults forage for chick provisioning and self-maintenance [28]. Before sunrise, little penguins aggregate and travel in groups to the water’s edge [29] and then spend between 14 and 18 h at sea per day [27]. When returning to the colony, conspecifics raft together in waters close to the shore before making landfall in groups after sunset [30,31]. Adults will continue to return to the colony this way in order to feed their young until they fledge, usually after eight or nine weeks [25]. This pre- and post-foraging behaviour is thought to be a predator avoidance strategy [32] where individuals are most vulnerable to predation from foxes, cats and avian predators when returning to land [33].

Recently, it has been documented that little penguins also display group behaviour at sea [19,34]. Individuals were found to synchronise their diving behaviour with conspecifics over a large portion of a foraging trip, potentially with benefits for detecting, aggregating or capturing prey and/or predator avoidance likely indicating cooperative foraging. However, these preliminary studies were limited to a small sample size and single foraging trips and, therefore, little is known about the factors influencing this behaviour and if individuals forage with the same partners over time. Furthermore, very little is known regarding group size during foraging activity aside from visual observations. A greater understanding of group foraging behaviour and group size may provide insight into factors influencing optimal group foraging.

Therefore, the aims of the present study were to determine in little penguins the: 1) factors influencing foraging associations; 2) temporal consistency of such associations; and 3) foraging group size.

## Materials and methods

### Ethics statement

The study was conducted in accordance with the guidelines of Deakin University Animal Ethics Committee (Approval B21-2013) and under the Wildlife Research Permit (10006877) from the Department of Environment and Primary Industries (Victoria). London Bridge in Port Campbell National Park and Gabo Island Lighthouse Reserve were accessed under permit from Parks Victoria.

### Study site, animal handling and instrumentation

**Fig 1:**
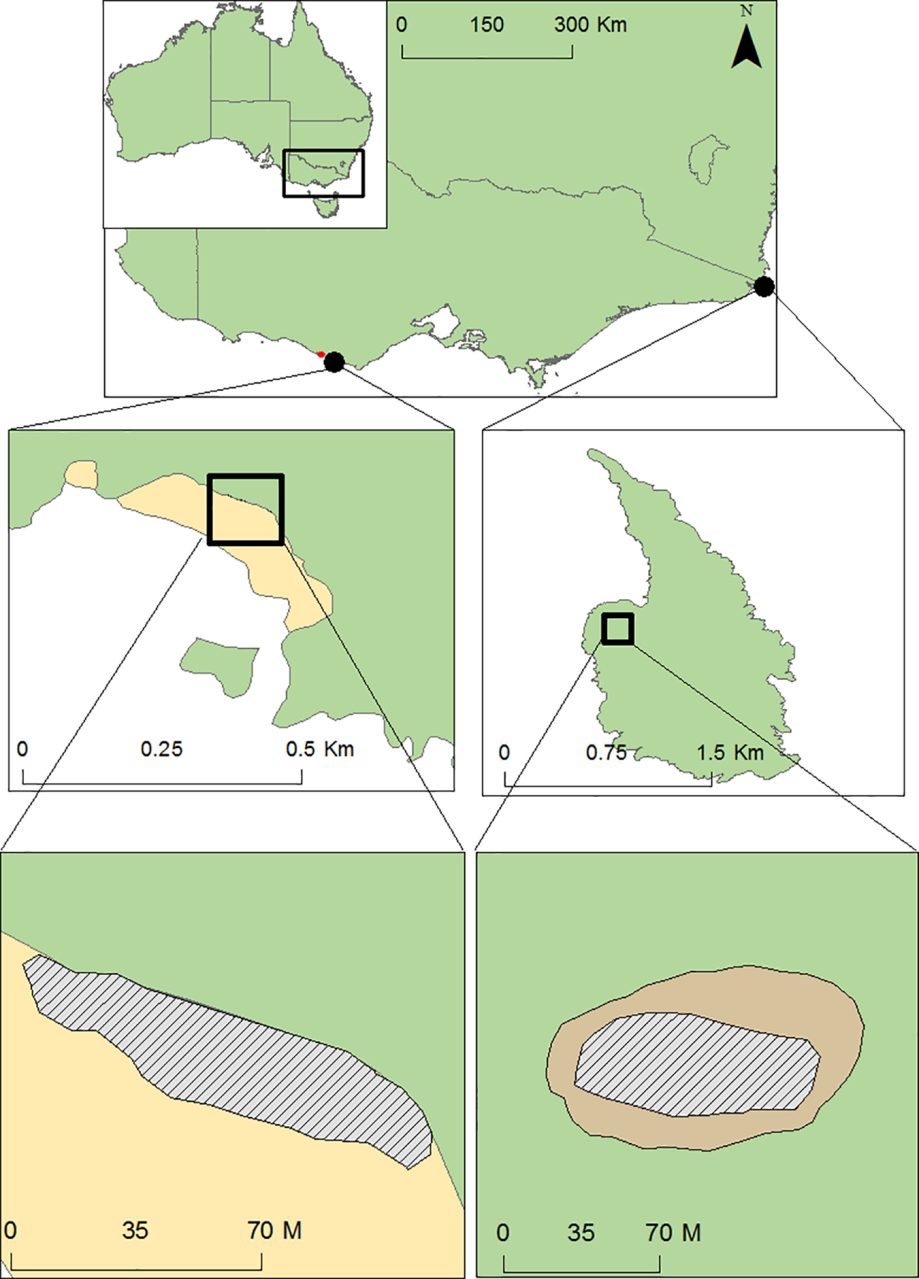
Location of London Bridge (left) and Gabo Island (right) along the south-east Australian coastline. Area sampled (hatched polygon) at Gabo Island was separated from the rest of the colony (grey polygon) by an area of open marshy field where there were no nests.

To assess the potential influence of colony size on foraging associations, the study was conducted at two breeding colonies in south-eastern Australia during the 2014–15 chick rearing period (August-January): London Bridge (LB; 38°33’S, 142° 55’E) in western Bass Strait and Gabo Island (GI; 37°33’S, 149°54’E) in eastern Bass Strait (Fig 1). The LB colony is a small colony (approximately 800 m2) located on the Australian mainland at the base of sandstone cliffs and, limited by suitable nesting habitat, is comprised of only 100–150 individuals. GI is a 1500 m2 island hosting 30–40,000 breeding individuals all over the island in high nesting densities along the coastal edges [35].

In order to assess the effect of nesting proximity on association behaviour, an area of similar size and nesting density of the small LB colony was chosen at GI. At GI, the area sampled was separated from the rest of the colony by a region of open marshy field unoccupied by nesting penguins due to the topography and poor drainage (Fig 1). Nesting density (nests·m^-2^) was measured by assessing occupancy of all the burrows in the chosen location and identifying those which were occupied with breeding individuals or chicks (indicating foraging adults). Distances between nests of associating individuals were measured using a tape measure (± 1 cm) and handheld GPS and distance from the middle of the high tide mark was measured using ArcMap to estimate how far individuals at each colony would have to walk to get to the ocean [36].

At each site, individuals from the sample nesting areas were randomly selected for deployment of data loggers. Breeding adults with one or two chicks were sampled during guard stage, where breeding partners alternate each day between foraging and remaining in the burrow to protect offspring; and post-guard stage, where both partners forage each day [25].

**Fig 2:**
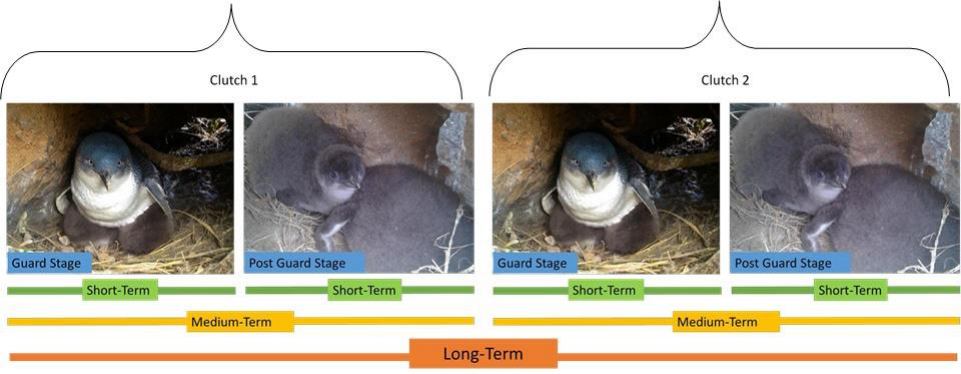
The temporal consistency of associations was investigated by sampling the same individuals over 3 time-scales: within chick rearing stages (*Short-term*); between chick rearing stages (*Medium-term*); and between clutches (*Long-term*).

Birds were captured in their nest burrows and weighed in a cloth bag with a spring balance (± 0.1 kg). Morphometric measurements were taken using Vernier callipers (± 0.1 mm): bill depth was measured for sexing; bill length, bill width, head length and flipper length were measured for indication of structural size; and body mass and flipper length to calculate a Body Condition Index [37–39]. Individuals were instrumented with a GPS data logger (IgotU GT120, Mobile Technologies; 5 cm x 3.5 cm, 12 g) which provided a fix every 2 min, a dive behaviour data logger (LAT 1500, Lotek Wireless; 3.2 cm x 0.8 cm, 3.4 g) programmed to sample dive depth every 4 s. The GPS and accelerometer were sealed together in heat shrink tubing for waterproofing and, along with the dive behaviour data logger, were attached to the feathers using black waterproof tape (Tesa, 4651, Beiersdorf, AG, GmbH, Hamburg). After 4 days the devices were removed, the bird was weighed and placed back into its nest to resume normal activity. Where possible, in order to investigate the temporal consistency of associations, the same individuals were sampled at 3 temporal scales: within chick rearing stages (S*hort-term*); between chick rearing stages (*Medium-term*); and between clutches (*Long- term*) (Fig 2). When an individual was sampled at more than one temporal stage, the tag was removed and re-deployed at each stage. To ensure the deployment of loggers on the correct individuals for subsequent stages, birds were implanted with a passive integrated transponder tags (PIT tag, 11 × 1.5 mm, Trovan), which were implanted in the subcutaneously above the scapulae at first capture [30].

Video data loggers (BirdCam v. 2, Catnip Technologies, 30 x 45 x 15 mm, 20 g, 854 x 400 pixels at 28–30 frames per second) and dive behaviour data loggers (as specified above) were deployed on additional individuals for a single foraging trip during the guard stage at both colonies. Video loggers were programmed to record footage for 15 minutes per hour to assess the potential for individuals associating with non-instrumented conspecifics. Together, the devices weighed 3% of body mass and were <0.03% of body cross-sectional surface area. Birds instrumented in the study showed similar foraging depths and time at sea to those instrumented with previous studies [27,30,40]. Therefore, the devices would have had negligible effects on the animals.

In addition to deployments, pre-foraging group formation of departing individuals was investigated using a night vision lens (Bushnell Monocular Night Vision, 2.5 x 4.2 mm, field of view: 24.08 m.). Observations were conducted for 7 mornings from the same location at each colony during the deployment period. Number of departing individuals, group size and a qualitative description of behaviour were recorded from 03:30 am local time until sunrise, approximately 3 hours later.

### Data processing and statistical analyses

Dive data were corrected for depth drift using the *diveMove* package [41] in R [42] and GPS locations were summarised following the application of a speed filter [43] using the *trip* package [44]. The GPS data were linearly interpolated at an interval of 4 seconds to estimate the location of each dive, and then individually separated into single foraging trips by removing time on land according to the wet or dry state of the dive behaviour data loggers.

Interpolated tracks were analysed using a custom-written script in Eonfusion (Myriax Pty. Ltd., Hobart, Australia). Following the methods of Berlincourt and Arnould [34], a foraging association was defined as 2 or more instrumented birds actively swimming and diving together within a radius of 500 m and for at least 748 s, which represent the average horizontal distance travelled and the mean dive bout duration during foraging activity. Rafting behaviour close to the colony, along with the inward and outward commuting phases close to the colony of the trip were visually determined from the interpolated data and excluded, limiting analyses to the foraging phase of the trip. A Linear Mixed-Effects model (LME) was used to compare the level of association between the two colonies. A response variable representing the number of associations for each individual foraging trip was fitted against a two-category predictor variable representing the two colonies. The model was fitted using the *nlme* package (v. 3.1–120) [45]. Associations between the same individuals over subsequent trips, stages or reproductive attempts were assessed by calculating the percentage of trips that individuals foraged with the same partners.

Generalised Linear Mixed Models (GLMM) [46], using a binomial error distribution with a logistic link function were used to determine the factors influencing the likelihood of association. A Bernoulli response variable represented association (1) or no association (0) between all possible pairs of individuals (121 possible pair combinations) at sea at the same time. This was fitted against the four predictor variables: distance between nests, sex of associating pair (same sex pair, different sex pair), the absolute difference between body condition (body mass divided by flipper length), and time difference of entering the water between all concurrently tracked individuals (log-transformed to improve model fit/normalise residuals), as well as the interaction between time entering the water and the sex of the associating pair. Same sex pair vs different sex pairs were also assessed using a three level factor within the model (male-male, female-female and male-female), the results of this model were consistent with the results of the same sex vs different sex model and showed a higher AIC (334 vs 322). As such, we only show the results of the model containing the grouping same sex pair vs different sex pair. Collinearity between predictor variables was assessed using pairwise correlations, variance inflation factors and visualisations (for factor, continuous pairings) and none was identified. The crossed random terms (individual bird IDs for each bird in the pair) were nested within the random term, dyad, comprised of all combinations of pairs of individuals that were completing a foraging trip at the same time. To assess the factors influencing the likelihood of associations, an information theoretic approach was adopted whereby combinations of the predictor set were ranked using Akaike’s Information Criterion corrected for small sample sizes (AICc). A subset of the most likely models (cumulative sum of AICc weights < = 0.95) was then used to generate model averaged coefficient estimates using the *MuMIn* package (ver 1.15.6) [47].

Tracks were inspected for incidences where an individual associated with the same bird over more than one foraging trip. Temporal consistency of re-associations was assessed at three temporal scales: Short-term—consistency in foraging with the same individuals over consecutive foraging trips; Medium-term—foraging with the same individuals between stage (guard and post-guard); and Long-term—foraging with the same individual between clutches.

In order to assess foraging group size, video footage from animal-borne video data loggers were viewed using VLC media player [48]. Presence of conspecifics was recorded in order to determine maximum group size. Maximum group size was defined as the maximum number of conspecifics in a single frame during the entire period at sea (including the camera bearer). The maximum foraging group size was determined for each individual from the video footage and averaged for each colony in order to make group size comparisons between colonies.

## Results

### At-sea associations

A total of 152 trips were obtained from 79 individuals instrumented with GPS and dive behaviour data loggers for between 2 and 5 foraging trips (Table 1). Foraging associations were observed in > 90% of instrumented individuals at LB during both guard and post-guard stages, significantly more than at GI where only 18 and 32% of instrumented birds associated for each stage respectively (Table 2). Individuals at LB associated with multiple instrumented individuals throughout their foraging trips, this was consistent across all breeding stages. Comparatively, GI individuals displayed significantly less associations with an estimated average of 1.17 less associations at GI than at LB (LME, F_1_ = 23.38, *P* < 0.001; Table 1). Body condition was only marginally higher at LB colony 10.24 ± 0.36 g/mm compared to GI 9.12 ± 0.17 g/mm. was calculated for both sites and was approximately the same (0.05 and 0.04 nests·m^-2^ at LB and GI, respectively).

**Table 1:** Deployment summary of little penguins (*Eudyptula minor*) instrumented with GPS and dive behaviour data loggers. **Birds instrumented in guard phase were re-instrumented in their following breeding stages in order to investigate temporal patterns.** A summary of the complete number of trips obtained, the mean number of consecutive trips per individual and the mean number of associations per trip per individual for each breeding stage at LB and GI colonies.

Colony	Stage	*N*	Sex		Complete trips obtained	Mean number consecutive trips per individual (range)	Mean number associations per trip per individual ± S.E.
			M	F			
LB	Guard	13	7	6	21	1.6 (1–2)	1.2 ± 0.3
	Post-guard	14	7	7	36	2.5 (1–4)	1.9 ± 0.4
	2^nd^ clutch Guard	11	6	5	22	2 (1–2)	1.7 ± 0.2
	2^nd^ clutch Post-guard	5	1	4	8	1.6 (1–3)	2.3 ± 1.0
GI	Guard	18	10	8	28	1.6 (1–3)	0.1 ± 0.1
	Post-guard	18	10	8	30	1.7 (1–4)	0.5 ± 0.1
Total		79	41	38	145		

**Table 2:** Percentage of instrumented individuals that associated with other instrumented conspecifics. A summary of the number of birds tracked simultaneously and the percentage of the total number of individuals associating at sea during the guard and post-guard phase at both colonies.

Colony	Stage	n	Individuals simultaneously tracked	Percentage of tracked individuals associating (%)
LB	Guard	24	5.4 ± 0.3	97.6
	Post-guard	19	6.9 ± 1.2	93.8
GI	Guard	18	7.5 ± 1.4	17.9
	Post-guard	18	7.3 ± 1.5	31.6

With the exception of a few individuals at the GI colony, individuals at both colonies remained within a 40 km range of the colony (S1 Fig). While the majority (>90%) of individuals at LB foraged with conspecifics, few individuals were found to associate at GI (4 and 7 out of 18 individuals for guard and post-guard stages, respectively). As such, factors influencing associations were investigated at LB only.

For LB, the mean difference in condition between associating pairs was 2.37 ± 0.25 g/mm and non-associating pairs was 2.87 ± 0.25 g/mm. The mean distance between nests of associating and non-associating pairs were remarkably similar being 16.24 ± 0.77 m and 16.86 ± 0.80 m, respectively. On average, associating individuals entered the water within 32.13 ± 2.74 minutes of each other while individuals who did not associate entered the water in 46.67 ± 2.47 minutes of each other (S5 Data).

**Fig 3:**
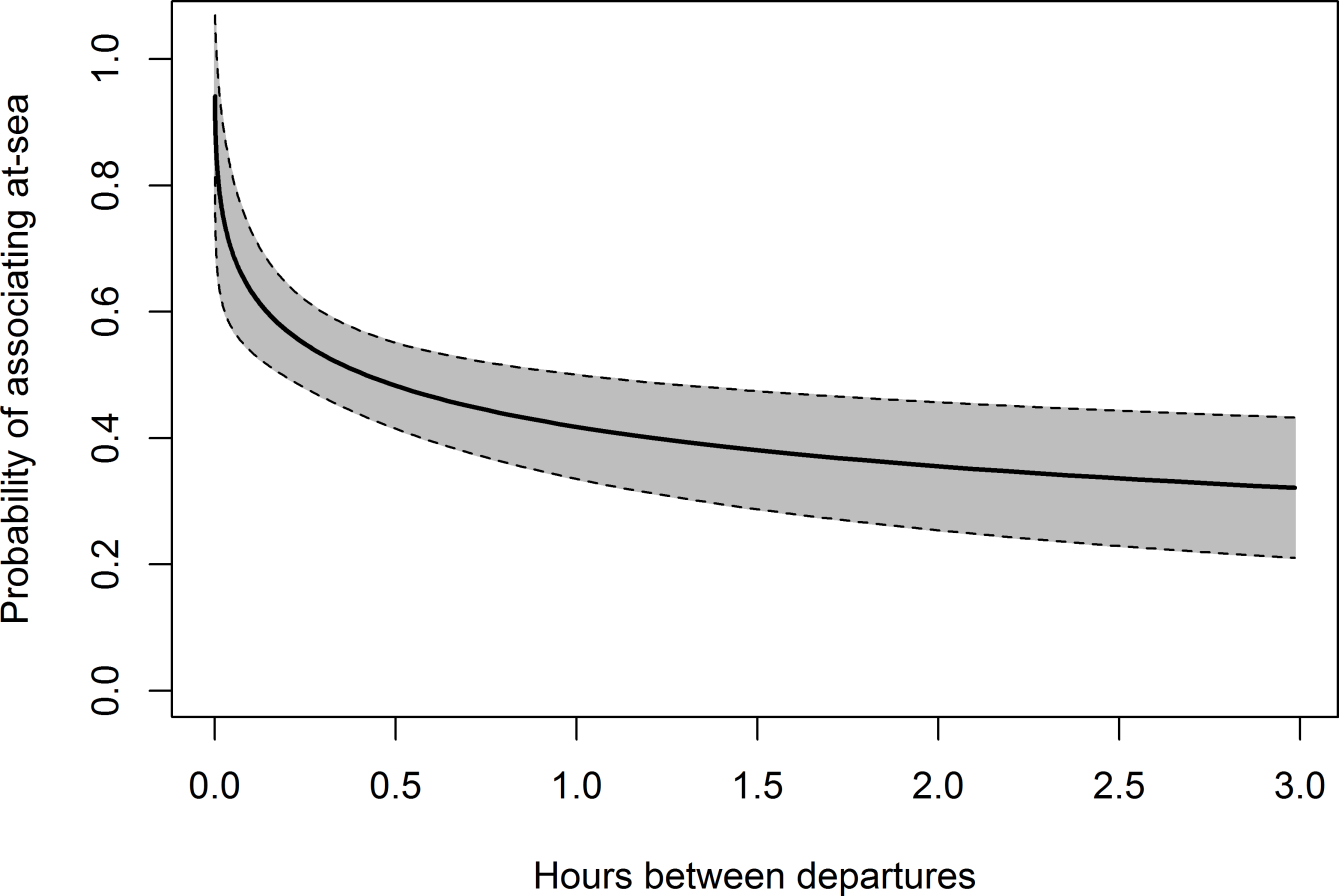
The predicted probability of little penguins from London Bridge colony associating at-sea as a function of the time between their departures from land. Grey shading shows the 95% confidence interval around the mean.

Ranking of each individual GLMM by AICc resulted in a subset of 9 out of 20 possible models remaining within the 0.95 threshold of the cumulative sum of AICc weights (Table 3; S1 Table). This final model set contained all four primary coefficients and the interaction term, however, only the coefficient for difference between departure times had unconditional confidence intervals whose values did not cross zero (Table 4). Profile likelihood confidence intervals for parameter estimates of individual models showed results consistent with the model-averaged results with only the difference between departure times parameter having confidence intervals that did not cross zero in all models. This suggests that the difference between departure times was the only variable where we were sufficiently confident to have detected an effect. The difference between departure times show a negative relationship with the probability of individuals associating at-sea, with the probability of individuals associating at-sea declining rapidly as the difference between their departure times increased (Fig 3; Table 4).

**Table 3:** Most likely models, in descending order, evaluating the probability that pairs of foraging little penguins will associate at sea.

Candidate models	df	LogLik	AIC_c_	ΔAIC_c_	AIC_c_ Wt
**Probability of associating at-sea ~**					
log(time between departure) + Sex of birds	8	-155.72	328.1	0.00	0.26
log(time between departure)	7	-157.03	328.6	0.48	0.20
log(time between departure) + Sex of birds + log(time between departure)*Sex of birds	9	-155.56	329.9	1.84	0.10
log(time between departure) + Sex of birds + Distance to nests	9	-155.57	329.9	1.86	0.10
log(time between departure) + Sex of birds + Individual condition	9	-155.64	330.1	2.00	0.09
log(time between departure) + Individual condition	8	-156.88	330.4	2.31	0.08
log(time between departure) + Distance to nests	8	-156.97	330.6	2.51	0.07
log(time between departure) + Sex of birds + Distance to nests + log(time between departure)*Sex of birds	10	-155.41	331.8	3.73	0.04
log(time between departure) + Sex of birds + Individual condition + log(time between departure)*Sex of birds	10	-155.46	331.9	3.83	0.04

LogLik = the log likelihood of the models, AIC_c_ Wt = The AIC_c_ Weight of each model

**Table 4:** Model averaged parameter estimates from models evaluating the probability that pairs of foraging little penguins will associate at sea. Bold parameter estimates represent those whose 95% unconditional confidence intervals did not cross zero.

Parameters	β^−^±SE(β^−^)	*z* value (*p* value)	95% *CI* *(lower–upper)*	Relative importance	*n models*
Intercept	**2.78 ± 1.01**	**2.64 (0.008)**	**0.71–4.86**		
log(time between departure)	**-0.38 ± 0.14**	**2.80 (0.005)**	**-0.65 –-0.11**	**1.00**	**9**
Sex of birds	-0.71 ± 1.01	0.69 (0.488)	-2.71–1.29	0.64	6
log(time between departure) * sex of birds	0.12 ± 0.23	0.56 (0.572)	-0.32–0.58	0.22	3
Distance to nests	-0.07 ± 0.15	0.47 (0.639)	-0.37–0.23	0.22	3
Individual condition	-0.09 ± 0.20	0.46 (0.643)	-0.49–0.31	0.18	3

### Temporal consistency in associations and pre-foraging group formation

Individuals at LB displayed a high consistency of associations with the same conspecifics across three temporal scales (Table 5; S2 Fig). As with single-trip associations, individuals at GI displayed almost no re-association. While there were no clear patterns of repeated associations, more repeated Short-term associations occurred in post-guard stage rather than the guard stage (LB Guard 1 = 2; LB Post-Guard 1 = 17 re-associations) as birds were departing on foraging trips every day rather than alternating days.

**Table 5:** Percentage of individuals that re-associate with the same conspecifics over 3 temporal scales: within stages *(Short-term*); between stages (*Medium-term*), and between stages (*Long-term*) where n is the number of individuals in each category. 64% of 43 individuals foraged with each other on more than one occasion during the Short-term. 53% of 15 individuals that were tracked in both Short and Medium-terms associated with the same conspecifics between stages (Medium-term) and 60% of 5 individuals tracked in the first clutch also associated with the same conspecifics in the second clutch (Long-term).

Colony	Percentage of re-association (%)		
	Short-term	Medium-term	Long-term
LB	64 *n* = 43	53 *n* = 15	60 *n* = 5
GI	0 *n* = 36	10 *n* = 10	*

*Birds at GI did not double clutch this season, hence, Long-term associations could not be assessed

**Fig 4:**
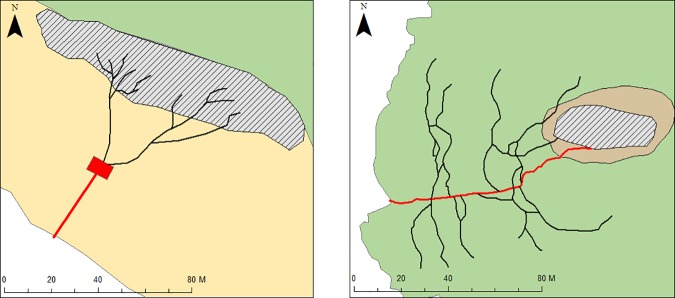
Pre-foraging group formation at LB (left) and GI (right). LB birds traversed pathways (black lines) from the study area (hatched polygon) to the staging area (red rectangle) from where they crossed the expanse to the ocean. At GI birds used a main pathway (red line), which was accessed via tributaries by many individuals throughout the colony.

Visual observations of instrumented and non-instrumented individuals departing the colony (*n* = 7 days at both sites; approximately 42 h) revealed differences in behaviour due to local topography and distance from the sea. Upon leaving their nest, individuals at LB traversed a short distance to a waiting area at the base of a dune, congregating with conspecifics approximately 23 m from the middle of the intertidal zone (Fig 4). Once a group of between 8 and 15 birds had gathered at this staging area in the cover provided by the dune vegetation, individuals walked together across the beach to the water. In contrast, individuals at GI walked along a distinct tortuous pathway, approximately 116 m long, leading to the water (Fig 4). This pathway was well worn into the vegetation with tributaries providing access to the pathway from other nesting locations beyond the study area in the colony. Consequently, instrumented individuals were observed to join with conspecifics from other breeding areas on the main pathway en route to the water. While individuals at GI also congregated in small groups prior to entering the water, this occurred along the main pathway where individuals from tributaries joined established groups.

At LB, birds travelled from their burrows to a meeting point and together, crossed to the water’s edge while individuals at GI used a main pathway to the shoreline. Individuals from other parts of the colony accessed this pathway via tributaries.

### Foraging group size

A total of 20 individuals (10 male, 10 female) were instrumented with video data loggers (Table 6) for a single foraging trip during guard-stage. Due to instances of device malfunction, not all devices recorded at the programmed duty cycle of 15 min recording 45 min off, instead, some individual periods of video data swapped the duty cycle and recorded for 45 min with 15 minutes off. Nonetheless, video data were obtained at intervals throughout the entire foraging trip (i.e. outward/inward commute, diving periods and resting at the surface) for an average of 3.6 ± 0.2 h, representing 26.7 ± 1.7% of the average foraging trip for both locations.

**Table 6:** Deployment summary for little penguins (*Eudyptula minor*) instrumented with video and dive behaviour data loggers. A summary of the number of birds tracked, the mean recording time of the cameras and the mean maximum number of individuals in observed in a group (including the camera bearer) at both colonies.

Colony	*n*	Sex		Mean recording time ± SE (h)	Mean max group size per trip ± SE
		M	F		
LB	10	5	5	3.5 ± 0.4	4.2 ± 1.3
GI	10	5	5	4.5 ± 0.4	7.5 ± 2.3

At both colonies, conspecifics were visible in the video data obtained. At LB, instrumented birds travelled and foraged with both other instrumented and non-instrumented conspecifics. Birds were seen travelling and capturing prey (small Clupeoid fish, Crustacea and jellyfish) in the presence and absence of conspecifics (see S1 and S2 Video in [19] for additional information). In the presence of conspecifics, individuals did not seem to work in any coordinated fashion, rather they seem to compete for prey or ignore conspecifics at the prey patch altogether. Group size varied throughout the foraging trip from solitary foraging to group sizes between 2 and 24 individuals with the maximum number of individuals observed at any one time being 24 and 17 at GI and LB, respectively. The mean maximum foraging group size was not significantly different between both colonies (Mann-Whitney’s *U* test, *U* = 70, *P* > 0.05).

## Discussion

In the present study, little penguins were found to associate at sea and on the shoreline in groups, displaying consistency with whom they associated with over multiple time-scales. While no relationships could be found between intrinsic factors and the level of association, results indicate that population size and pre-foraging group formation may influence the formation of both temporary and consistent foraging associations, suggesting association behaviour may be opportunistic in nature.

### Intrinsic and extrinsic factors influencing foraging associations

Sexual selection has led to the development of sexual dimorphism where one sex, usually male, is larger in size [49]. Consequently, each sex may have different metabolic requirements particularly during the breeding season, which could lead to a degree of dietary segregation [50]. Forming groups according to sex may minimise potential energetic trade-offs [51]. On one hand, it may be advantageous for individuals of different sexes to forage together, to avoid competition at the same prey patch [52,53]. On the other hand, it may benefit individuals of the same sex to forage together to reduce the metabolic cost of maintaining group cohesion [54]. In little penguins, males are not only heavier, but also possess slightly longer flippers and larger bill depths than females [37]. As a result, males have the ability to swim faster, dive to greater depths for longer periods and are thought to consume larger prey [55,56]. In the current study, there was no apparent influence of sex on foraging associations. It is possible that inter sexual dive differences could occur at a small scale, however, based on the video data, individuals are finding prey in locations where it is accessible to both males and females. As males can only dive slightly deeper than females [30], in years where prey availability is low, sex may play a role in group formation, however this was not the case in this study.

Synchronous group behaviour between individuals of different sizes may be energetically costly due to a dissimilarity in activity budgets [54]. Consequently, groups may form due to similarities in body condition [57]. Many species, including the little penguin, are known to form groups based on condition [29,54,58] and according to similar age [29]. It has been suggested that in years of high breeding success, group formation on land between little penguins of similar quality or age may reflect group dynamics at sea [29]. In the present study, the influence of age on associations was not investigated as tracked individuals were not of a known age and, therefore, age-related factors during pre-foraging group formation could not be addressed. Furthermore, there was no apparent influence of body condition on foraging associations. The LB colony is located near the seasonally active Bonney Upwelling, which makes the surrounding waters highly productive [59] and may explain the generally high Body Condition Index and higher incidence of double clutching observed here in comparison to other sites [27]. In contrast to previous studies, individuals in the present study were of similar body condition with birds from LB having slightly higher ratios than GI (S3 Data). Furthermore, there was very little variation in body condition between individuals at the LB colony. Therefore, it is not possible to discount the potential influence of these factors on the formation of foraging associations.

The *information centre hypothesis* proposes that individuals at a colony directly or indirectly broadcast explicit cues to colony members regarding the location or type of prey in the environment [8]. Therefore, it might be expected that individuals are more likely to exchange information regarding resources with conspecifics that nest in close proximity over those that nest further away. Cues such as chicks begging/being fed or individuals of good physical condition may indicate foraging ability. However, consistent with the findings of the preliminary study by Berlincourt and Arnould [34] birds nesting directly next to each other were not more likely to associate than birds nesting at a greater proximity to each other.

### Influence of pre-foraging group formation and population size

Congregation may be the mechanism which social interaction and cooperation ensues [60]. For example, little penguins assemble in groups on land and offshore upon departing and returning from a foraging trip. Group association in little penguins may provide individuals with protection as well as increase their ability to find and aggregate prey [19]. The benefits of repeated associations may include the ability to predict the foraging decisions of conspecifics that an individual regularly forages with thereby increasing the success of a foraging group. This certainly occurs in individuals that develop long-term associations, such as dolphins that develop techniques for catching small fish over time [61]. However, in the present study, departure time was found to influence associations, as individuals who left at similar times were more likely to associate. This may suggest that little penguins are opportunistically associating with their surrounding conspecifics and could decide upon their foraging partners based on the immediate pool of conspecifics that depart at the same time. Therefore, pre-foraging group formation, where individuals assemble on land in groups before entering the water, may provide insight into how population size and the topography of the nesting site may influence association behaviour. During the early morning observations of individuals leaving the colony, behaviours were observed at both colonies, where differences in terrain determined pre-foraging group formation.

Individuals at the small LB colony congregate to cross an exposed beach in small groups in order to access the water. This group formation may also facilitate birds meeting up with conspecifics from all over the colony, thereby fostering high levels of associations irrespective of influencing factors. In comparison, due to a limited capacity for terrestrial locomotion in dense vegetation [62], penguins at GI followed a series of interconnecting tributaries which merged onto to a well-defined pathway leading down to the water’s edge. These tributaries act to “funnel” individuals from all over the colony towards the main pathway, resulting in interactions between neighbour and non-neighbour individuals. The large numbers of birds simultaneously accessing the pathways caused dilution of the instrumented birds into the rest of the population. This resulted in significantly fewer associations between instrumented conspecifics.

In a small colony, such as LB, individuals are more likely to meet familiar conspecifics due to small population size and, consequently, it is possible that associations are formed based on previous outcomes of foraging together. This behaviour may account for the high degree of temporal associations at LB observed in the present study. Alternatively, since individuals are restricted to a limited number of conspecifics with whom they can associate, group associations, which appear to have a high temporal consistency, may be by chance and simply be due to a limited number of potential partners. Since most individuals leave the colony within a narrow time window (before sunrise), the probability of the same individuals meeting over numerous occasions at the staging area is high. In contrast, individuals in the larger GI colony have many more potential conspecifics with whom to associate. While group associations could be random or opportunistic, pre-foraging group behaviour at GI may allow birds from separate nesting areas in the colony to form long-term associations.

### Optimal foraging: group size and consistency

While a low level of association was recorded at GI between individuals instrumented with GPS and dive behaviour data loggers, video data from this colony revealed animals regularly associated with groups of conspecifics. These results indicate that associations recorded by individuals instrumented with GPS and dive behaviour data loggers are an under-estimation of the prevalence of group foraging behaviour at both colonies.

If group capacity is above what is optimal for individuals to achieve ideal prey intake, it is expected for individuals to defect from the group as association with conspecifics becomes costly [12,63]. Animals must consider the costs and benefits of certain strategies to make decisions about whether or not they should be repeated [64]. The ability to retain information about prior events is essential for individuals to decide whether or not to continue to cooperate [11]. Food abundance, distribution and the presence of conspecifics are the key determinants of foraging behaviour [10,58]. Sutton et al. [19] found that little penguins equipped with video camera and GPS experienced no calorific benefits when hunting a variety of prey types in groups compared to alone. While individuals may not seem to be working in a coordinated fashion, it is possible that the presence of multiple individuals at a prey patch is keeping the fish aggregated near the surface. This may suggest a potential trade-off between an increased chance of finding prey, and hunting success.

In summary, the results of the present study have demonstrated that little penguins may show consistency in association behaviour. While no relationships were found between sex, nest proximity, and the degree of association, population size and tributary path was found to influence the level of neighbourly associations. In the small LB colony, birds are limited in choice and there may be fitness benefits to foraging consistently with the same individuals. In the large GI colony, video data loggers revealed that individuals also associated with conspecifics while foraging. While they displayed a comparatively low level of associations and re-associations with their proximate neighbours, it is unknown whether these individuals are re-associating with the same conspecifics from other parts of the colony. At the GI colony, individuals have a larger pool of potential foraging partners and a longer period while walking to the water and we hypothesise that they may be forming associations during this period. At the smaller LB colony, it may be less likely for individuals to encounter each other at sea, while at the larger GI colony, it is perhaps more likely for individuals to encounter each other at sea. Therefore, in order to attain the benefits of group foraging, LB penguins must congregate leaving the colony.

While the present study revealed that time leaving the colony affected the likelihood of association, it is unclear if little penguins actively select foraging partners during departure from the colony. Instead, individuals could be opportunistically assessing potential foraging partners during pre-foraging group formation or it may be that individuals are departing the colony at the same time and, consequently, foraging together. Furthermore, video data revealed consistent patterns of group association from both colonies, which may indicate an optimal group foraging size for hunting small schooling prey and, thus, influence association patterns.

## Supporting information

S1 Data Little penguin foraging association modelling data. (CSV)

S2 Data Early morning observations. Observations were conducted for a total of 7 nights from the same location at each colony during the deployment period.
(XLSX)

S3 Data Condition of all individuals used in the study. (XLSX)

S4 Data Nest locations of all individuals used in this study. Nests were marked with handheld GPS (±3m accuracy).
(XLSX)

S1 Table Full list of models, in descending order, used for assessing the probability that pairs of foraging little penguins will associate at sea. (DOCX)

S2 Table Summary statistics for associating and non-associating pairs at LB colony. (DOCX)

S1 Fig All GPS tracks obtained from 2014–15 breeding season. (TIF)

S2 Fig Representative re-associations. **Each colour represents an individual and thick lines indicate periods of association.** Short-term (left): Two individuals associating over consecutive trips; Medium-term (left and middle): the same individual (blue) that associated in guard stage, clutch 1 also associated in post-guard stage, clutch 1 and Long-term: the same individuals associated during clutch one (middle) and clutch two (right).
(TIF)
