# Supplementary material for: Departure time influences foraging associations in little penguins
Source: PLoS One. 2017 Aug 23;12(8):e0182734. doi: 10.1371/journal.pone.0182734 (PMC5567918; doi:10.1371/journal.pone.0182734)
Supplement: S1 Table — (DOCX) [file pone.0182734.s005.docx]

**S1 Table: Full list of models, in descending order, used for assessing the probability that pairs of foraging little penguins will associate at sea**

| **Candidate models** | **df** | **LogLik** | **AIC_c_** | **∆AIC_c_** | **AIC_c_ Wt** |
| --- | --- | --- | --- | --- | --- |
| **Probability of associating at-sea ~** |  |  |  |  |  |
| Intercept + log(time between departure) + Sex of birds | 8 | -155.72 | 328.1 | 0.00 | 0.26 |
| Intercept + log(time between departure) | 7 | -157.03 | 328.6 | 0.48 | 0.20 |
| Intercept + log(time between departure) + Sex of birds + log(time between departure)*Sex of birds | 9 | -155.56 | 329.9 | 1.84 | 0.10 |
| Intercept + log(time between departure) + Sex of birds + Distance to nests | 9 | -155.57 | 329.9 | 1.86 | 0.10 |
| Intercept + log(time between departure) + Sex of birds + Individual condition | 9 | -155.64 | 330.1 | 2.00 | 0.09 |
| Intercept + log(time between departure) + Individual condition | 8 | -156.88 | 330.4 | 2.31 | 0.08 |
| Intercept + log(time between departure) + Distance to nests | 8 | -156.97 | 330.6 | 2.51 | 0.07 |
| Intercept + log(time between departure) + Sex of birds + Distance to nests + log(time between departure)*Sex of birds | 10 | -155.41 | 331.8 | 3.73 | 0.04 |
| Intercept + log(time between departure) + Sex of birds + Individual condition + log(time between departure)*Sex of birds | 10 | -155.46 | 331.9 | 3.83 | 0.04 |
| Intercept + log(time between departure) + Sex of birds + Distance to nests + Individual condition | 10 | -155.50 | 331.96 | 3.89 | 0.03 |
| Intercept + log(time between departure) + Distance to nests + Individual condition | 9 | -156.82 | 332.43 | 4.36 | 0.03 |
| Intercept + log(time between departure) + Sex of birds + Distance to nests + Individual condition+ log(time between departure)*Sex of birds | 11 | -155.32 | 333.82 | 5.75 | 0.01 |
| Intercept | 6 | -163.33 | 339.03 | 10.96 | 0.00 |
| Intercept + Sex of birds + | 7 | -162.53 | 339.54 | 11.47 | 0.00 |
| Intercept + Individual condition | 7 | -162.98 | 340.46 | 12.38 | 0.00 |
| Intercept + Distance to nests | 7 | -163.17 | 340.83 | 12.76 | 0.00 |
| Intercept + Sex of birds + Distance to nests | 8 | -162.25 | 341.14 | 13.07 | 0.00 |
| Intercept + Sex of birds + + Individual condition | 8 | -162.26 | 341.16 | 13.09 | 0.00 |
| Intercept + Distance to nests + Individual condition | 8 | -162.83 | 342.29 | 14.22 | 0.00 |
| Intercept + Sex of birds + Distance to nests + Individual condition | 9 | -162.00 | 342.80 | 14.73 | 0.00 |

LogLik = the log likelihood of the models, AIC_c_ Wt = The AIC_c_ Weight of each model
