# Supplementary material for: Departure time influences foraging associations in little penguins
Source: PLoS One. 2017 Aug 23;12(8):e0182734. doi: 10.1371/journal.pone.0182734 (PMC5567918; doi:10.1371/journal.pone.0182734)
Supplement: S2 Table — (DOCX) [file pone.0182734.s006.docx]

| **S2 Table: Summary statistics for associating and non-associating pairs at LB colony** | | |
| --- | --- | --- |
|  | Associating pairs | Non-associating pairs |
| Number of same sex pairs | 57.00 | 57.00 |
| Number of different sex pairs | 102.00 | 73.00 |
| Mean distance between nests (m) | 16.24 ± 0.77 | 16.86 ± 0.80 |
| Range (m) | 0 - 49 | 0 - 49 |
| Mean condition between pair ± SE (g/mm) | 2.68 ± 0.25 | 2.66 ± 0.25 |
| Range (g/mm) | 0 - 19.58 | 0 - 19.58 |
| Mean leaving time between pair ± SE (min) | 32.13 ± 2.74 | 46.67 ± 2.47 |
| Range (min) | 0.8 - 129.6 | 0 - 179.4 |
