# Supplementary figures and images for: Departure time influences foraging associations in little penguins
Source: PLoS One. 2017 Aug 23;12(8):e0182734. doi: 10.1371/journal.pone.0182734 (PMC5567918; doi:10.1371/journal.pone.0182734)

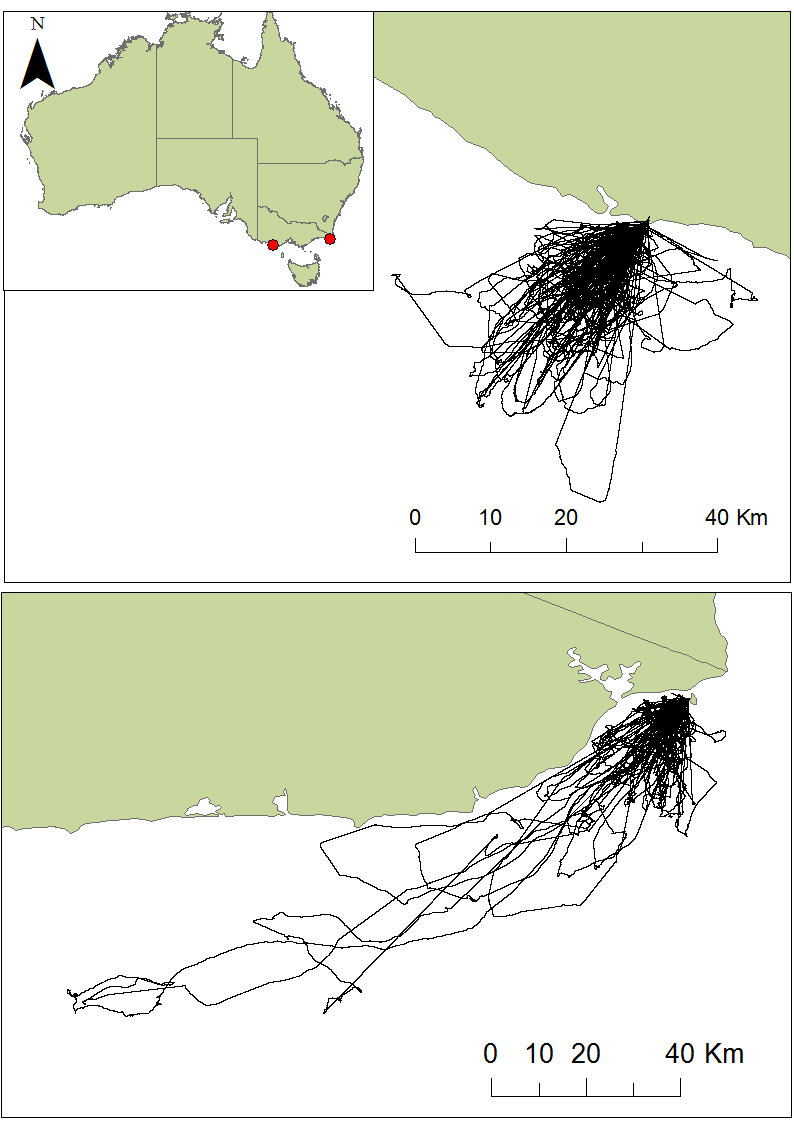

Supplement: S1 Fig — (TIF) [file pone.0182734.s007.tif]

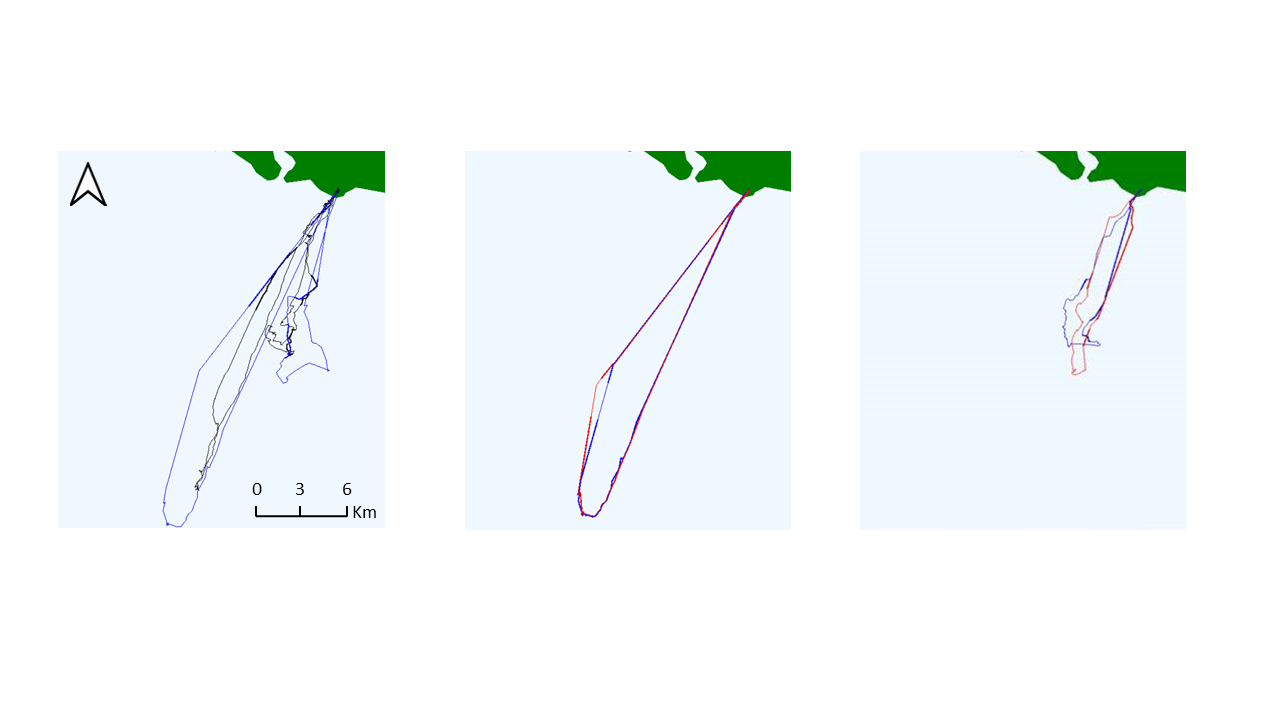

Supplement: S2 Fig — Each colour represents an individual and thick lines indicate periods of association. Short-term (left): Two individuals associating over consecutive trips; Medium-term (left and middle): the same individual (blue) that associated in guard stage, clutch 1 also associated in post-guard stage, clutch 1 and Long-term: the same individuals associated during clutch one (middle) and clutch two (right). (TIF) [file pone.0182734.s008.tif]
